# Hydropic anthelmintics against parasitic nematodes

**DOI:** 10.1371/journal.ppat.1008202

**Published:** 2020-01-30

**Authors:** Satish Kumar Rajasekharan, Jintae Lee

**Affiliations:** School of Chemical Engineering, Yeungnam University, Gyeongsan, Republic of Korea; University of Wisconsin Medical School, UNITED STATES

## Cellular hydrodynamics and swelling

**Fig 1:**
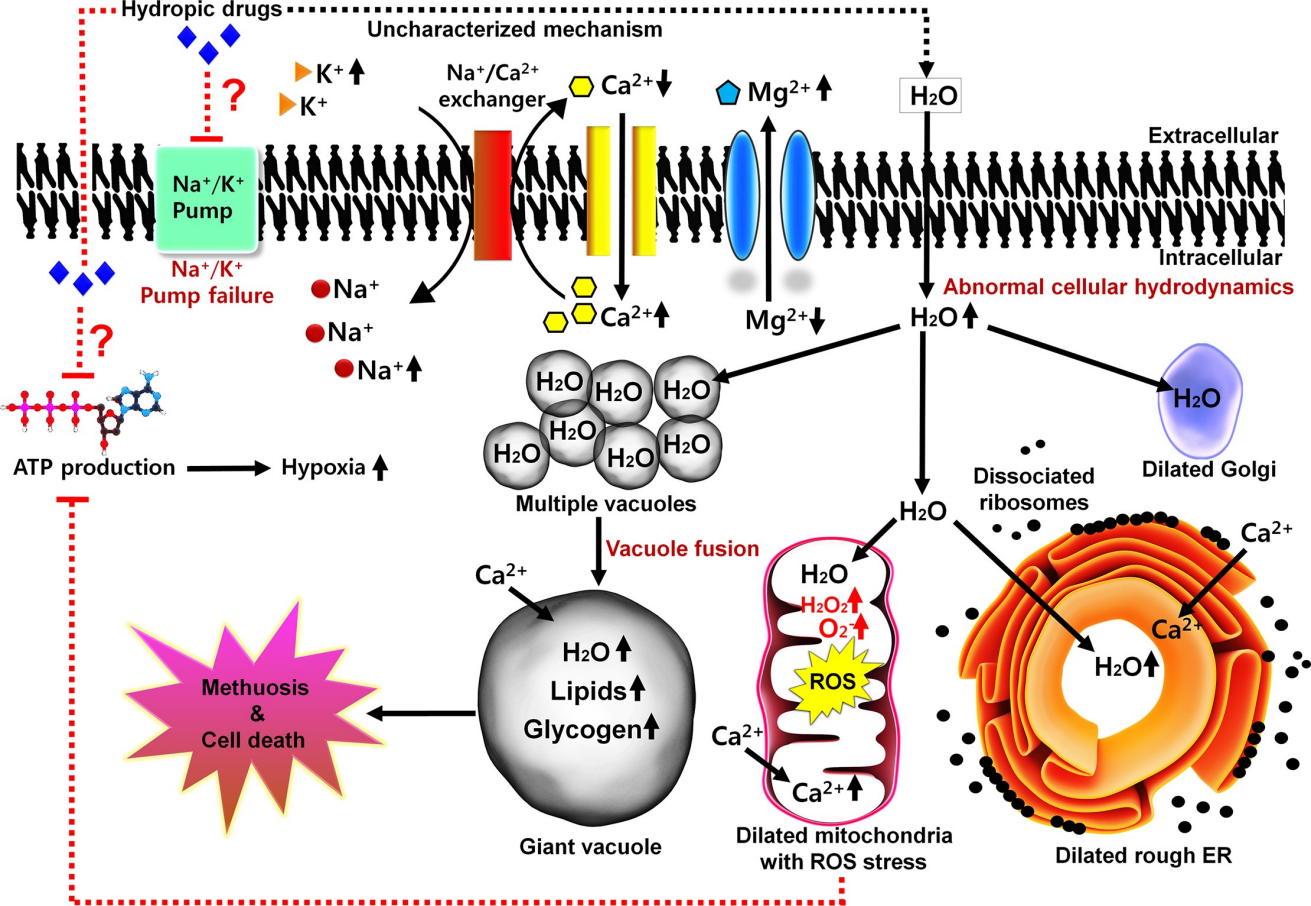
Biochemical changes that occur in a dying cell with disrupted osmoregulation. It is believed that hydropic drugs might inactivate the Na^+^/K^+^ antiporter pump or alternatively lead to decreased ATP production, ultimately leading to accumulation of Na^+^, Ca^2+^, and water and effluxes of K^+^ and Mg^2+^. A decreased Mg^2+^ level also suggests impaired functioning of the Na^+^/K^+^ pump. The drug might cause a rapid influx of water by an as of yet unknown mechanism, causing organelles (vacuoles, Golgi bodies, mitochondria, and rough endoplasmic reticulum) to dilate extensively and disrupting normal biological functioning. Water-filled vacuoles tend to fuse to form giant vacuoles that subsequently rupture and kill cells. Contrariwise, mitochondrial damage might also be a factor for decreased ATP production in the cell. The dashed lines (red or black) indicate uncharacterized sections of the pathway, while solid lines (black) represent regular biological attributes. ATP, adenosine triphosphate; ER, endoplasmic reticulum; ROS, reactive oxygen species.

Hydrodynamic forces that typically act in healthy cells maintain fluid osmoregulation by balancing hydrostatic and oncotic pressures [1]. The sodium–potassium pump has a fundamental role in the maintenance of cellular osmoregulation [2]. Functionally, the sodium–potassium pump is regulated by Na^+^/K^+^ adenosine triphosphate (ATP)ase, a ubiquitous enzyme that continually uses ATP by pumping 3 Na^+^ ions out of and importing 2 K^+^ ions into cells. When a constant sodium gradient is upheld, water molecules cross the plasma membrane by passive diffusion, maintaining dynamic homeostasis. Failure of the Na^+^/K^+^ ATPase pump to regulate homeostasis results in membrane depolarization and eventually cellular swelling and death [2]. Any form of stress or injury to the membrane or the molecular pump adversely affects cell-resting potential and causes rapid Na^+^, Ca^2+^, and water influx. The water influx causes vacuoles, mitochondria, rough endoplasmic reticulum, and Golgi bodies to dilate and deteriorate, and under these circumstances, small vacuoles fuse to form giant vacuoles (Fig 1), which might be a hindrance to cell convalescence. Overall, abnormal water ingress disrupts normal cell hydrodynamics and eventually stimulates cell-death pathways.

## Archetypes of cell death

Cell death is generally defined by comparing idiosyncratic morphological or phenotypic anomalies with standard references to biochemical and/or signal transduction pathways [3]. The fate of a dying cell solely depends on the intensity and duration of the stress or injury it has incurred. If an injury is mild and transient, cells can acclimatize and recover, in the absence of additional stress and given sufficient nutrients and ATP. Recoverable injuries are often described as reversible cell injuries [3]. Nevertheless, unless overly severe, cells can revert to normality, provided the cause of the injury is removed or the cell is pharmacologically treated. In contrast, irreversible cell injury occurs when injury or stress is severe, progressive, and irretrievable [4]. Such an affected cell passes an irreversibility threshold point and experiences a permanent and rapid loss of function, accompanied by the collapse of cellular organelles, plasma membrane, and chromatin. Although the cellular manifestations of both types of injuries share analogous topographies in the initial stages, a cell injury can shift from reversible to irreversible. Following the discovery of watery vacuoles in liver cells in 1946 [5], several reports have confirmed the occurrence of these water bodies inside several other cell types exposed to chemical or environmental stressors [6]. Cell death induced by the accumulation of vacuoles is indeed unlike apoptosis, necrosis, or autophagy. Methuosis, oncosis, paraptosis, and necroptosis are closely related death processes and are classified as types of nonapoptotic cell death induced by the aggregation of vacuoles [6]. Methuosis is a canonical form of vacuolar cell death and the most recent addition to the array of cell-death phenotypes. Basically, cells undergoing methuosis are characterized by a sequence of anomalies, resulting in the formation of large lucent cytoplasmic vacuoles.

**Fig 2:**
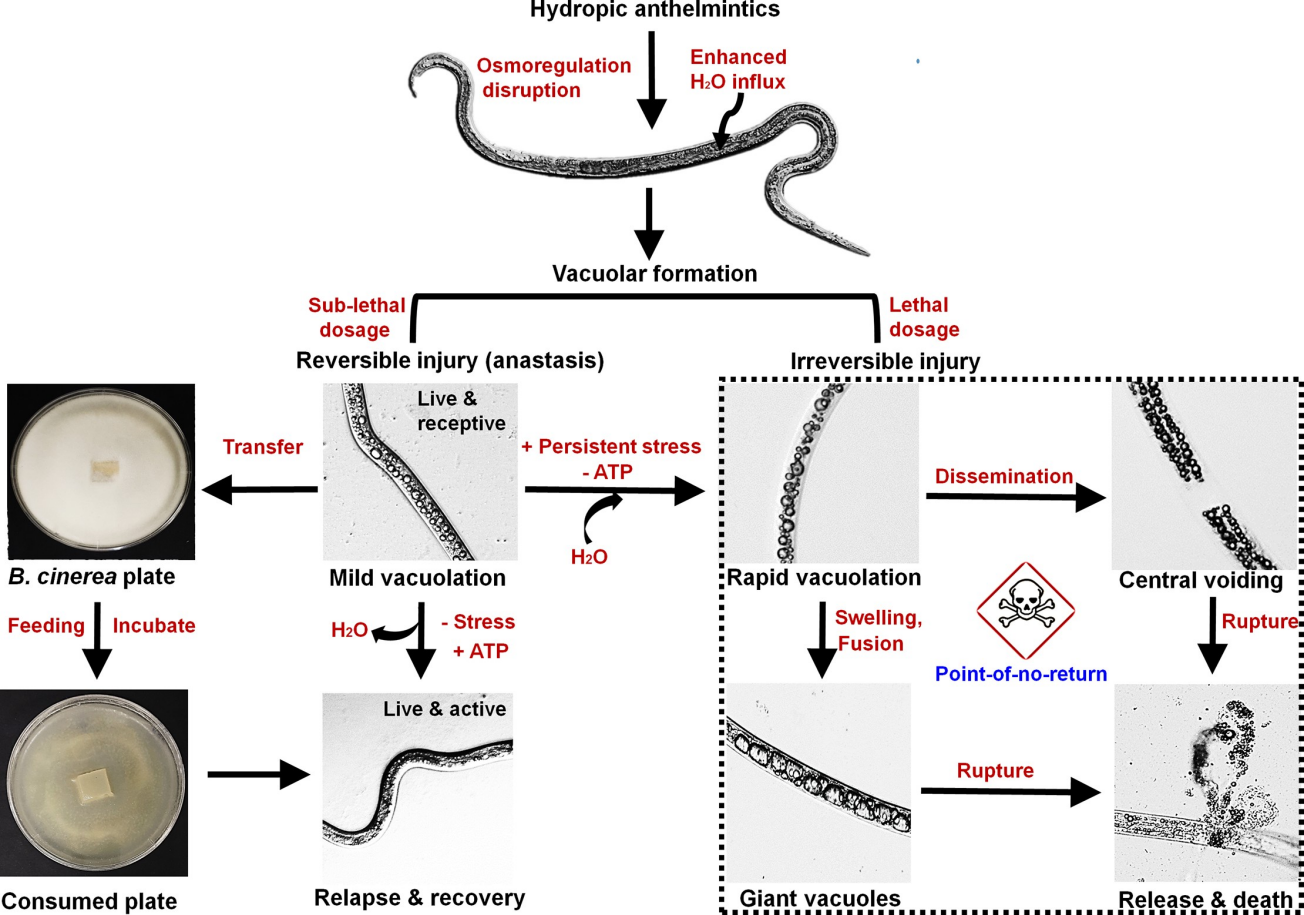
Sequence of vacuolar cell death in parasitic nematodes. Hydropic anthelmintics, such as 5-iodoindole, disrupt osmoregulation causing rapid water influx into nematodes. This leads to the formation of multiple vacuoles. Depending on the severity of the injury or chemical dosages, injury due to vacuolization can be classified as reversible (where the nematodes can be revived back to life) or irreversible (where the nematodes pass the point of no return, causing them to die). ATP, adenosine triphosphate. The phenotypes were re-constructed by adopting images and concepts from our previous publications [12, 14].

The nematode is a multicellular worm that contains a fluid-filled body cavity called the pseudocoelom, which bathes its internal organs. The organs consist of mouth, pharynx, alimentary, and the reproductive and the excretion systems [7]. Methuotic death in nematodes is marked by sequence of events that arise due to tissue death or organ damages. Importantly, the vacuolar phenotypes formed are distinctly visible through their body (Fig 2), confirming the involvement of multiple cells. Vacuolar death in nematodes might also be closely associated with abnormal pseudocoelom, although it is yet to be determined.

## Chemicals that induce vacuoles in nematodes

The appearance of lipid droplets or vacuoles has been reported in nematodes when administered with certain chemicals, which herein we categorize as hydropic anthelmintics. Trademark fertilizers, like Calphos (calcium phosphonate) and Magphos (magnesium and potassium phosphonate), were first shown to cause vacuole formation in the intestines of *Meloidogyne incognita* and *M*. *javanica* [8]. Seo and colleagues reported the occurrence of vacuoles in the mid and posterior regions of *M*. *incognita* J2 treated with mixtures of lactic and acetic acids and hypothesized the involvement of cell and tissue disruptions, as previously reported when *Caenorhabditis elegans* was exposed to heat-shock conditions [9]. Also, Jang and colleagues reported that the vacuolar phenotypes induced by oxalic acid and other acids may be due to osmotic imbalance leading to fluid accumulation [10].The first study to provide valuable insights regarding the composition of vacuoles was conducted by Bogneret and colleagues, who described the emergence of vacuole-like droplets in the central and tail regions of *M*. *incognita* J2s (as explained by Hasbash and colleagues) treated with indole-3-acetic acid or 4-hydroxybenzoic acid extracted from *Fusarium oxysporum* 162 [11]. This group also showed that the droplets predominantly contained glycerophospholipids and sphingolipids, polyketides, prenol lipids, and glycerolipids [11]. In their report, they suggested the occurrence of these lipid droplets, which subsequently fuse, is a biological marker of parasite death [11]. As reported by that group, *M*. *incognita* J2 death under experimental conditions is inevitable as J2s do not feed in an external environment. While upholding the same observations for *M*. *incognita* J2, we have alternatively shown that feeding can revive a different nematode (*Bursaphelenchus xylophilus*) from vacuolar death [12]. In addition, we have confirmed the presence of progressive death by vacuolation in all instar stages of *B*. *xylophilus* and in *M*. *incognita* J2 stages following 5-iodoindole treatment. Spectinabilin (a nitrophenyl-containing polyketide) is the only other chemical reported to induce vacuolar death in *B*. *xylophilus* [13].

## Vacuolar death in a nematode model

Methuotic or vacuolar death in nematodes (a phenotype characterized by lopsided vacuolization) is often characterized by the formation of multiple vacuoles and their subsequent fusion to form giant vacuoles [12]. Although seldom studied, this form of death is usually reversible but may be irreversible under adverse conditions. Based on our observations of *B*. *xylophilus* phenotypes after treatment with sublethal (0.05-mM) or lethal (0.1- or 0.2-mM) dosages of 5-iodoindole, we developed a proposed overview of the pathway to survival or lethality (Fig 2) [12]. At sublethal dosages, 5-iodoindole induces small vacuoles on nematodes, but its supplementation with a fungal feed (*Botrytis cinerea*) allows surviving nematodes to recover [12,14]. These observations suggest that arresting vacuolar swelling by providing an energy source (ATP) can effectively avert nematode death. Alternatively, if the cause of stress is persistent or progressive, the nematodes enter a death phase (passing a point of no return or point of irreversibility) characterized by the appearance of dilated or giant vacuoles, which eventually swell and rupture.

Understanding the chemistry and structure of a nematicidal drug is an obligatory part of the design and synthesis of novel drugs. Indole-based chalcones have been shown to induce methuosis in U251 glioblastoma (GBM) cells and in other cell lines [15]. It is reported that *para* positioning of nitrogen in the pyridinyl moiety of chalcones is vital for vacuolization, which is abolished when the nitrogen was at the *meta* position [6]. In addition, positioning of the methoxy group at the fifth position of indole in indolyl–pyridinyl–propenone analogs induced methuosis, while shifting the methoxy group to the sixth position abolished methuosis and disrupted microtubules in GBM cells [16]. Similarly, 2 of the iodoindoles (5-iodoindole and 7-iodoindole) have elicited vacuoles in nematodes. Although both of them had similar effects at high dosages, 5-iodoindole was more potent and achieved the phenotype at concentrations 10-fold lower than that of 7-iodoindole. The data reflect the positioning of iodine on the fifth position of indole ring, which also has a significant role in the induction of methuosis, as reported by Maltese and colleagues [6]. Meanwhile, other research groups have also provided sufficient evidence that other chemicals can elicit vacuolar death in nematodes and thus have opened a new possibility for drug discovery. Thus far, the compounds that triggered vacuolar death in nematodes seem to possess a halogenated atom (iodine or fluorine) with an indole moiety or a hydroxyl group (-OH) along with a mono- or di-carboxyl group (-COOH) (Table 1). It is interesting to note that indole-3-acetic acid, oxalic acid, 4-hydroxybenzoic acid, lactic and acetic acids are closely related and all of them possess either a mono- or di-carboxyl group. Acids might create an osmotic imbalance within the cells causing fluid accumulation which might be presented as vacuoles. Understanding the dynamics and exact principle of acid-induced fluid accumulation will indeed assist to screen -COOH functional group containing chemicals that are eco-friendly and cost-effective. Indole derivatives were the first ones shown to induce methuosis. In nematodes, the derivatives with iodine groups elicited the vacuolar phenotypes [12]. Although the exact biochemical question of how the process is driven is not addressed, our in vitro data suggest that the iodine–indole complex is a key driving factor for vacuolization. Synthetizing or repurposing newer drugs that contain the iodine and indole complex is one of starting points for hydropic anthelmintics.

**Table 1:** Chemicals that trigger vacuole formation in parasitic nematodes.

Chemicals	Functional group(s)/atoms	Target parasite	Phenotypes	Concentrations	Stages	Reference
Acetic acid	-COOH	*M*. *incognita*	Multiple vacuoles	0.1%–1%	J2	[9]
Calphos	N/A	*M*. *incognita* *M*. *javanica*	Large vacuoles	0.5 and 1%	J2	[8]
Indole-3-acetic acid	-COOH	*M*. *incognita*	Vacuole-like droplets	117 μg/mL	J2	[11]
Lactic acid	-COOH	*M*. *incognita*	Multiple vacuoles	0.5%	J2	[9]
Magphos	N/A	*M*. *incognita*, *M*. *javanica*	Large vacuoles	0.5%	J2	[8]
Oxalic acid	HO_2_C−R−CO_2_H	*M*. *incognita*	Multiple vacuoles	2 mM (180.06 μg/mL)	J2	[8]
Spectinabilin	−NO_2_, = O, -CH_3_, and O–CH_3_	*B*. *xylophilus*	Multiple vacuoles	0.84 μg/mL	Adult	[10]
4-Hydroxybenzoic acid	-COOH	*M*. *incognita*	Vacuole-like droplets	104 μg/mL	J2	[11]
5-Iodoindole	Iodine	*B*. *xylophilus*, *M*. *incognita*	Methuosis	0.1 mM (24.3 μg/mL)	Instar stages, adult, and eggs	[12,14]
7-Iodoindole	Iodine	*B*. *xylophilus*	Giant vacuoles	1 mM (243 μg/mL)	Instar stages, adult, and eggs	[12]

-COOH, carboxyl group; N/A, Not applicable

## Urge for screening new candidate classes of anthelmintics

Drug resistances in human or veterinary nematodes is not a major issue at present; however, if drugs are used spasmodically, the problem might arise [17]. Resistances to macrocyclic lactones, imidazothiazoles, benzimidazoles, and levamisole have recently been reported in several nematodes that infect goats, horses, sheep, and cattle. Most anthelmintics, like macrolactones and levamisole, function by paralyzing the nematodes [18]. Laboratory experiments have shown nematodes paralyzed with drugs can be revived back to normality, depending on the drug dosage used [18]. It is imperative to screen or repurpose eco-friendly drugs that kill nematodes without harming the host. For instance, few vacuole-inducing chemicals, such as spectinabilin, 5-iodoindole, 4-hydroxybenzoic acid, and indole-3-acetic acids, have been shown to have very low or weak toxicities to mammalian cell lines or mice models. Spectinabilin is an established antimalarial and antiviral agent [19], and 4-hydroxybenzoic acid is a popular antioxidant used in topical and parenteral pharmaceutical products or cosmetics [20]. Our experiments with 5-iodoindole revealed that the chemical was not toxic to plants and mice models [14]. All these compounds are highly lethal to plant-parasitic nematodes while not to other eukaryotic models, and thus they can be effectively tested on human or veterinary nematodes. The data also specify that the compounds are probably targeting nematode receptor proteins. The exact mode of action and selectivity is still not clear, but several proposals arise. Hydropic drugs might inactivate the Na^+^/K^+^ antiporter pump or alternatively decrease ATP production directly or indirectly by compromising the mitochondrial function (Fig 1). Overall, repurposing chemicals as hydropic drugs that are effective against environmental nematodes on medically important mammalian-parasitic nematodes necessitate safety testing and targeted drug delivery managements in hosts.

## Conclusion

Therapeutic approaches that utilize methuosis in nematodes have not been adopted as of yet, and thus information on the subject is limited. Understanding the mechanism of vacuolization in parasitic nematodes will undoubtedly modify perceptions of the dynamic behavioral and resistance patterns of parasites. In conclusion, this opinion encourages scientists to synthetize, repurpose, or search for as of yet unknown vacuole-inducing chemicals and drugs that trigger water ingress and vacuole formation as apparent endpoint marker.
